# Xanthohumol hinders invasion and cell cycle progression in cancer cells through targeting *MMP2, MMP9, FAK and P53* genes in three-dimensional breast and lung cancer cells culture

**DOI:** 10.1186/s12935-023-03009-2

**Published:** 2023-08-02

**Authors:** Zohreh Gholizadeh Siahmazgi, Shiva Irani, Ali Ghiaseddin, Parviz Fallah, Vahid Haghpanah

**Affiliations:** 1 Department of Biology, Science and Research Branch, Islamic Azad University, Tehran, Iran; 2 Department of Biomedical Engineering Division, Chemical Engineering Faculty, Tarbiat Modares University, Tehran, Iran; 3 Laboratory Science Department, Allied Medicine Faculty, Alborz University of Medical Sciences, Karaj, Iran; 4 Endocrinology and Metabolism Research Center, Endocrinology and Metabolism Clinical Sciences Institute, Tehran University of Medical Sciences, Tehran, Iran; 5 Personalized Medicine Research Center, Endocrinology and Metabolism Clinical Sciences Institute, Tehran University of Medical Sciences, Tehran, Iran; 6 Endocrinology and Metabolism Research Center (EMRC), Dr. Shariati Hospital, North Kargar Ave, 14114 Tehran, Iran

**Keywords:** Xanthohumol, 3D cell culture, Spheroids, Anti-cancer agent

## Abstract

**Background:**

Despite recent advances in the treatment of lung and breast cancer, the mortality with these two types of cancer is high. Xanthohumol (XN) is known as a bioactive compound that shows an anticancer effect on cancer cells. Here, we intended to investigate the anticancer effects of XN on the breast and lung cancer cell lines, using the three-dimensional (3D) cell culture.

**Methods:**

XN was isolated from *Humulus lupulus* using Preparative-Thin Layer Chromatography (P-TLC) method and its authenticity was documented through Fourier Transform Infrared spectroscopy (FT-IR) and Hydrogen Nuclear Magnetic Resonance (H-NMR) methods. The spheroids of the breast (MCF-7) and lung (A549) cancer cell lines were prepared by the Hanging Drop (HD) method. Subsequently, the IC_50s_ of XN were determined using the MTT assay in 2D and 3D cultures. Apoptosis was evaluated by Annexin V/PI flow cytometry and *NFκB1/2*, *BAX, BCL2*, and *SURVIVIN* expressions. Cell cycle progression was determined by *P21*, and *P53* expressions as well as PI flow cytometry assays. Multidrug resistance was investigated through examining the expression of *MDR1* and *ABCG2*. The invasion was examined by *MMP2, MMP9*, and *FAK* expression and F-actin labeling with Phalloidin-iFluor.

**Results:**

While the IC_50s_ for the XN treatment were 1.9 µM and 4.74 µM in 2D cultures, these values were 12.37 µM and 31.17 µM in 3D cultures of MCF-7 and A549 cells, respectively. XN induced apoptosis in MCF-7 and A549 cell lines. Furthermore, XN treatment reduced cell cycle progression, multidrug resistance, and invasion at the molecular and/or cellular levels.

**Conclusions:**

According to our results of XN treatment in 3D conditions, this bioactive compound can be introduced as an adjuvant anti-cancer agent for breast and lung cancer.

## Background

Cancer is among the most critical health issues in the world wide [1]. Although many advances have been made in recent years in the treatment of various types of cancer, especially breast and lung cancer, there is still no promising treatment for them [2].

Xanthohumol (XN) is an important bioactive compound in the inflorescence of *Humulus lupulus*, which has been studied for its anti-cancer effects in two-dimensional (2D) cell cultures [3]. Some evidence has shown that XN can inhibit Mitogen-activated protein kinase/ Extracellular signal-regulated kinase (MAPK/ERK) pathway in cancer cells. The ERK/MAPK signaling pathway is involved in regulating cellular biological functions, such as cell proliferation, cell cycle regulation, and cell apoptosis [4].

Undoubtedly, the use of laboratory animals has always had a pivotal role in the development of medical science [5]. However, due to differences in human and laboratory animal biochemistry, the use of animals has limitations [5, 6]. On the other hand, in 2D cell culture, because of reduced cell contact, they cannot properly mimic tumor behaviors [7, 8]. To deal with this problem, researchers have developed three-dimensional (3D) cell culture. This system better mimics the physiological features of in vivo and provides important information about cell-cell interactions, as well as helps to introduce new small molecules [9]. Spheroids, as a 3D cell culture method, are known as microtumors, one of the best models for mimicking micrometastasis. Spheroids can be used to study the invasion, growth, and proliferation of tumors as well as the screening of drugs [7]. Using the hanging drop (HD) method, spheroids are formed at the same time and with the same size [6].

The purpose of the current study was to investigate the effect of XN as a natural compound on the cell cycle, apoptosis, invasion, and drug resistance under 3D conditions on homospheroid model of A549 and MCF-7 cell lines.

## Materials and methods

### Preparation of ethanolic extract

The wild *Humulus lupulus* plant was collected from the north of Iran (Guilan province). Female flower of *Humulus lupulus* was extracted by maceration method [10]. Briefly, 10 g of the crushed female flowers of *Humulus lupulus* were macerated in 100 ml of ethanol and shaken for 24 h. The extract was filtered with Whatman No: 1 filter paper.

### Preparative -thin layer chromatography (P-TLC)

Purification of XN from *H. lupulus* extract was carried out by analytical and preparative P-TLC. The solvent contained chloroform and methanol (8:2 v/v). Two solvents were first incubated for 24 h at room temperature to saturate. Next step, the extract (10 g) was coated on silica gel (Merck, Germany) column (20 cm×20 cm). The photos were taken by Canon camera using UV-transluminator (UV 254/312(365) - Iran). The retention factor of the spot was calculated [11].

### Fourier transform infrared spectroscopy (FT-IR)

The extracted material was completely dried and 1 mg of that substance was pressed as a pellet with 300 mg of potassium bromide by pressing machine (ParkinElmer, USA). FT-IR spectra analysis of XN showed that from the wavelength of 450 cm^− 1^ to 4000 cm^− 1^, peaks were obtained, which indicated the presence of functional groups on XN. The peaks were analyzed by referring to the Pavia book and the items in the IRPal2.0 software library.

### Hydrogen nuclear magnetic resonance (H-NMR)

H-NMR of XN was carried out by Bruker UltraShield (USA) at 300 MHz, using a 2.5 mm inverse-detection microprobe (z-gradiant).

### 2D cell culture

Human breast cancer cell line (MCF-7) and lung carcinoma cell line (A549) were purchased from Iranian biological resource center. Since the purpose of this study is to investigate the effect of important genes that are affected by the dysregulated ERK/MAPK pathway, the two selected cell lines had defected in the pathway.

The cells were seeded in Dulbecco's Modified Eagle’s Medium (DMEM) high glucose (Bioidea, Iran), 10% Fetal Bovine Serum (FBS) (Gibco, Canada), 1% penicillin and streptomycin (Gibco, Canada) at 37 °C and 5% CO_2_. Then, the cells were rinsed using phosphate-buffered saline (PBS) (Sigma-Aldrich, Germany), and detached with 0.25% trypsin/EDTA (Gibco, Canada).

### 3D cell culture

Spheroids were generated by HD technique. Cell density was calculated by Neobaure slide. The lids of 80 mm tissue culture dishes were inverted and then spotted with an array of 30 µl drops containing 25,000 cells (at least 50 drops per dish lid). Before placing back the lids on the tissue culture dishes, 10 ml sterile PBS was poured at the bottom of dishes to provide sufficient humidity in the chambers. The culture dishes were then incubated at 37^°^C and 5% CO_2_ for 24, 48, and 72 h. The formation of spheroids were evaluated daily by an inverted microscope. Moreover, spheroids’ sizes were measured by ImageJ software and their normality was examined by histogram drawing and statistical analysis in SPSS 2013 software.

### Observation of spheroids by scanning electron microscope (SEM)

Spheroids were fixed with 2.5% glutaraldehyde (Merck, Germany) in 0.1 M phosphate buffer (pH 7.2) for 4 h. Spheroids were washed by PBS and then dehydrated via graded series of ethanol (60%, 70%, 80%, 90% and 100% ethanol) (Merck, Germany). Finally, spheroids were coated with 1000 Å gold and evaluated under SEM (TESCAN VEGAII, Czech).

### Cytotoxicity assay in 2D cell culture

MTT [3-(4,5-dimethylthiazolyl-2)-2,5-diphenyltetrazolium bromide] assay for 2D cell culture was carried out according to the Mosmann method [12]. First of all, the MCF-7 and A549 cell lines were cultured in 96-well plates at a concentration of 5000 cells/well. Briefly, after 24, 48 and 72 h treatment with XN (300 µM, 150 µM, 75 µM, 37.5 µM, 18.75 µM and 9.37 µM), 20 µl of MTT solution (Sigma-Aldrich, Germany) was added to every well and incubated at 37 °C and 5% CO_2_ for 3 h. 150 µl of media was removed from each well of 2D cell culture plates and mixed with 100 µl of dimethyl sulfoxide (DMSO) (Sigma-Aldrich, Germany). Finally, optical density was recorded at 570 nm by an ELISA reader (Awareness, UK).

### Cytotoxicity assay in 3D cell culture

MTT assay for 3D cell culture was performed based on Ho et al. protocol [13] with insignificant modifications to monolayer cell culture. Spheroids were transferred to Ultra low attachment (ULA) 96 well plate (Corning, USA). Briefly, after 24, 48, and 72 h treatment with XN (300 µM, 150 µM, 75 µM, 37.5 µM, 18.75 µM and 9.37 µM), 20 µl of MTT solution (Sigma-Aldrich, Germany) was added to every well and incubated at 37 °C and 5% CO_2_ for 3 h. Plates were centrifuged at 1000×g for 5 min. 150 µl of media was removed from each well of 3D cell culture plates and mixed with 100 µl of dimethyl sulfoxide (DMSO) (Sigma-Aldrich, Germany). Finally, optical density was recorded at 570 nm by an ELISA reader (Awareness, UK).

All test assays were performed in 3D conditions.

### Invasion assay

Phalloidin-iFluor 488 (Abcam, UK) was used to evaluate the effect of XN on pseudopodia formation. After treatment with half-maximal inhibitory concentration (IC_50_) of XN (48 h), the spheroids (Treat and Untreat) were washed with 0.1 M PBS (pH 7.2), and fixed with 4% paraformaldehyde in PBS at room temperature for 20 min. The spheroids were rinsed 2 times in PBS. 0.1% Triton X-100 in PBS was added into fixed spheroids for 5 min to make them permeable and then the spheroids were blocked with 1% FBS for 20 min at room temperature. Next, spheroids were incubated with Phalloidin-iFluor 488 (Abcam, UK) and washed with PBS. Spheroids were rinsed gently with PBS two times to remove excess Phalloidin conjugate. Cells’ nuclei were stained with DAPI (Sigma-Aldrich, Germany) for 5 min. Finally, spheroids were analyzed with a fluorescence microscope (Laborlux D, Leitz, Germany).

### Cell cycle assay

First, the spheroids were treated with IC_50_ value of XN in ULA flasks for 48 h (Corning, USA). The Spheroids were washed using PBS. The spheroids were dissociated into single cells using TrypLE [14] (Gibco, Canada) and then TrypLE was neutralized by FBS. Finally, the cells were analyzed through (Propidium iodide (PI)) Kit (IQ Products, Netherlands) according to manufacturers’ instruction. Cell cycle progression of the cells was evaluated by flow cytometry using BD FACSCalibur. Data from cell cycle experiments were analyzed by FlowJo 7.6.1 software.

### Apoptosis assay

After treatment of the spheroids with IC_50_ of XN for 48 h in ULA flasks (Corning, USA), spheroids were transformed into single cells by TrypLE (Gibco, Canada) [14]. Apoptosis of XN-treated and XN-untreated spheroids was evaluated by AnnexinV (IQ Products, Netherlands) staining according to manufacturers’ protocol. The apoptotic cells were detected and quantified by flow cytometry using BD FACSCalibur. Data from apoptosis experiments were analyzed by FlowJo 7.6.1 software.

### Quantitative real-time PCR (qRT-PCR)

XN-treated and XN-untreated spheroids were harvested. Total RNA was extracted by TRIzol (SINACLON, Iran). The concentration of RNA was measured using a UV spectrophotometer (Thermo Scientific, UK). Then, cDNA was synthesized using a cDNA synthesis kit (SINACLON, Iran) based on the manufacturer’s protocol. RT-qPCR was carried out in the final volume of 20 µl which contained 10 µl SYBER Blue HS-qPCR (2×) (SINACLON, Iran), 2 µl cDNA, 1 µl forward and 1 µl reverse primers (*P53*, *P21*, *BAX*, *MDR1*, *NFkB1, NFkB2, SURVIVIN, FAK, MMP2, MMP9, MCL1, ABCG2, BCL2*) (Table 1). *β-ACTIN* was utilized as the reference gene for normalization. The qRT-PCR conditions were as follows: initial denaturation at 95 °C for 10 min, followed by 40 cycles for amplification (Second denaturation 95 °C for 10 s, annealing 60 °C for 30 s, and extension 72 °C for 30 s). The expression of genes was analyzed through the ABI StepOnePlus Real-Time PCR system (Applied Biosystems, USA).

**Table 1:** Sequences of the primers used in qRT-PCR

Gene name	Product length(bp)	Primer sequence
*P53*	181	F: GGAGTATTTGGATGACAGAA R: GATTACCACTGGAGTCTT
*P21*	150	F: CCAGCATGACAGATTTCTA R: AGACACACAAACTGAGACTAAG
*BAX*	187	F: CAAACTGGTGCTCAAG R: CACAAAGATGGTCACGGT
*MDR1*	161	F: TGGACAAGCACTGAAAGATAA R: TTCCTCAAAGAGTTTCTGTATG
*NFkB1*	182	F: GTGCTGGAGTTCAGGATAAC R:GTGGATGATTGCTAAGTGTAAGA
*NFkB2*	223	R: GAGTTGCTACAACCCAGGT F: ACAGTGGGATAGGTCTTTC
*SURVIVIN*	144	F: GACCACCGCATCTCTACAT R: CTCCTTGAAGCAGAAGAAAC
*FAK*	228	F: ATAGAACTTGGACGATGTATT R: TGACGCATTGTTAAGGCTT
*MMP2*	136	F: GCTCGTGCCTTCCAAG R: AGTCCGTCCTTACCGTC
*MMP9*	116	F: CGGACCAAGGATACAGTTT R: CTCAGTGAAGCGGTACATA
*ABCG2*	166	F: AACTCAGTTTATCCGTGGT R: CTGCTTAGACATCCT TTTCA
*BCL2*	70	F: GATAACGGAGGCTGGGA R: CAGGAGAAATCAAACAGAGG
*β-ACTIN*	215	F: CTTCCTTCCTGGGCA R: GTCTTTGCGGATGTCCA

*ABCG2* ATP-binding cassette subfamily G member 2, *BAX* Bcl-2-associated X protein, *BCL2* B-cell leukemia/lymphoma 2 protein, *FAK* Focal adhesion kinase, *MDR1* ATP Binding Cassette Subfamily B Member 1, *MMP2* Matrix metalloproteinase 2, *MMP9* Matrix metalloproteinase 9, *NFκB1* Nuclear Factor Kappa B Subunit 1, *NFκB2* Nuclear Factor Kappa B Subunit 2,* P2* Cyclin Dependent Kinase Inhibitor 1 A,

*P53* Tumor Protein P53

**Table 2:** The structure of XN and the FT-IR spectrum of extracted XN from *Humulus lupulus*

Wavenumber range (cm-1)	Types of bond	Chemical structure of XN
3400–3700	O-H	
2850	C-H	
1720–1740	C = O	
1000–1300	C-O	
1640–1680	C = C	
1000	C-C	
1000	C-O-C	
1650–2000		

*XN* Xanthohumol

*ABCG2* ATP-binding cassette subfamily G member 2, *BAX* Bcl-2-associated X protein, *BCL2* B-cell leukemia/lymphoma 2 protein, *FAK* Focal adhesion kinase, *MDR1* ATP Binding Cassette Subfamily B Member 1, *MMP2* Matrix metalloproteinase 2, *MMP9* Matrix metalloproteinase 9, *NFκB1* Nuclear Factor Kappa B Subunit 1, *NFκB2* Nuclear Factor Kappa B Subunit 2, *P21* Cyclin Dependent Kinase Inhibitor 1 A, *P53* Tumor Protein P53, *TFs* Transcription Factors

### Statistical analysis

The results of MTT assay were analyzed by One-way ANOVA (n = 4 per experiment). IC_50_ values were calculated by nonlinear regression for which GraphPad Prism V.8 software was employed. Statistical significances of gene expressions were determined using the unpaired t-test method by SPSS 2013 and relative expression levels were calculated using the 2^−ΔΔCt^ method (n = 3 per experiment). The data were presented as the mean ± standard error of the mean (SEM), *p* < 0.05 (*), *p* < 0.01 (**), *p* <0 .001 (***), *p* < 0.0001(****). Data from cell cycle and apoptosis experiments were evaluated by two-way ANOVA (n = 3 per experiment).

## Results

### **Isolation and verification of XN from the*H. lupulus*crude extract**

**Fig. 1:**
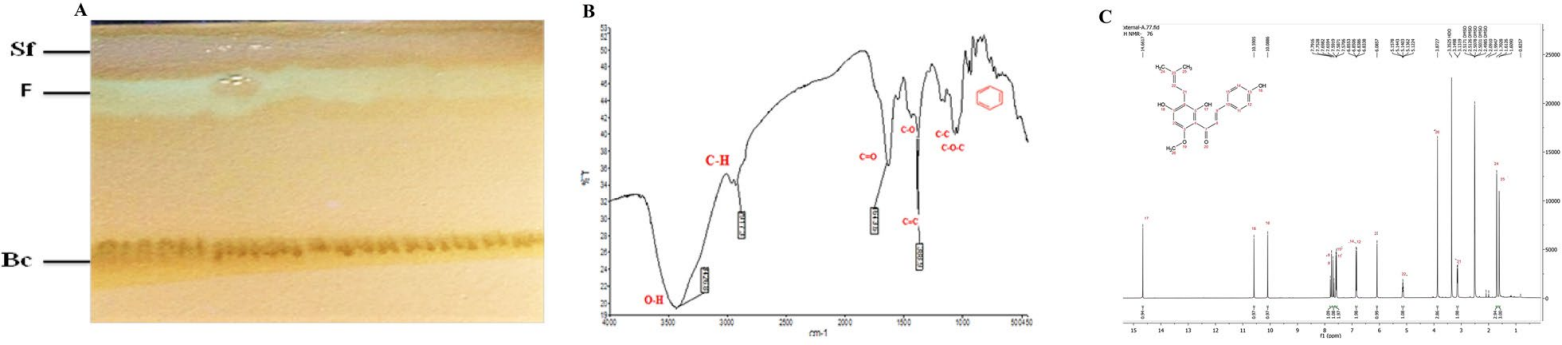
Isolation of XN from the *H. lupulus* crude extract.** A** P-TLC chromatography image shows a separated fraction containing XN fraction (F), baseline (BC), and a solvent fraction (Sf). **B** Verification of XN identity obtained from the *H. lupulus* fraction using the FTIR method. The graph shows the FT-IR curve for the isolated XN. Accordingly, O-H, C-H, C = O, C-O, C = C, C-O-C, and the aromatic ring in XN functional groups are the expected wave numbers in this diagram. **C** Verification of extracted XN identity through the H-NMR method. According to the radio frequencies, XN–specific peaks emerged following the application of the H-NMR method in which criteria were as follows: H-NMR (300 HZ): H: 1.61 (3 H, s), 1.70 (3 H, s), 3.14 (2 H, d, *J* = 5.37), 3.87 (3 H, s), 5.14 (1 H, t, *J* = 5.37), 6.09 (1 H, s), 6.85 (2 H, d, *J* = 5.01), 7.58 (2 H, d, *J* = 5.01), 7.68 (1 H, AB_4_, *J* = 9.31), 7.77 (1 H, AB_4_, *J* = 9.31), 10.09 (1 H, s), 10.59 (1 H, s), 14.66 (1 H, s)

The best solvent system for xanthohumol isolation was obtained chloroform/methanol (8:2 v/v) with a retention factor of 70%. The corresponding XN bond is present in the related silica gel plate (Fig. 1A). For Each 10 g of extract, 10 mg of XN was extracted, and the XN identity was verified using FT-IR and H-NMR. According to the IRPAL 2.0 software, the wavelength range for the OH, CH, C = O, C-O, C-C functional groups present in XN, was 3700 − 3400, 2850, 1740 − 1720, 1300 − 1000, 1680 − 1640, respectively. Aromatic ring was identified at 1650–1650 wavelength range (Fig. 1B; Table 2). H-NMR results were evaluated by Masternova software and confirmed based on Chen et al.[15]. The H-NMR spectra of the isolated compound are as follows (Fig. 1C): H-NMR (300 HZ): H: 1.61 (3 H, s), 1.70 (3 H, s), 3.14 (2 H, d, *J* = 5.37), 3.87 (3 H, s), 5.14 (1 H, t, *J* = 5.37), 6.09 (1 H, s), 6.85 (2 H, d, *J* = 5.01), 7.58 (2 H, d, *J* = 5.01), 7.68 (1 H, AB_4_, *J* = 9.31), 7.77 (1 H, AB_4_, *J* = 9.31), 10.09 (1 H, s), 10.59 (1 H, s), 14.66 (1 H, s).

### Spheroid formation by HD

**Fig. 2:**
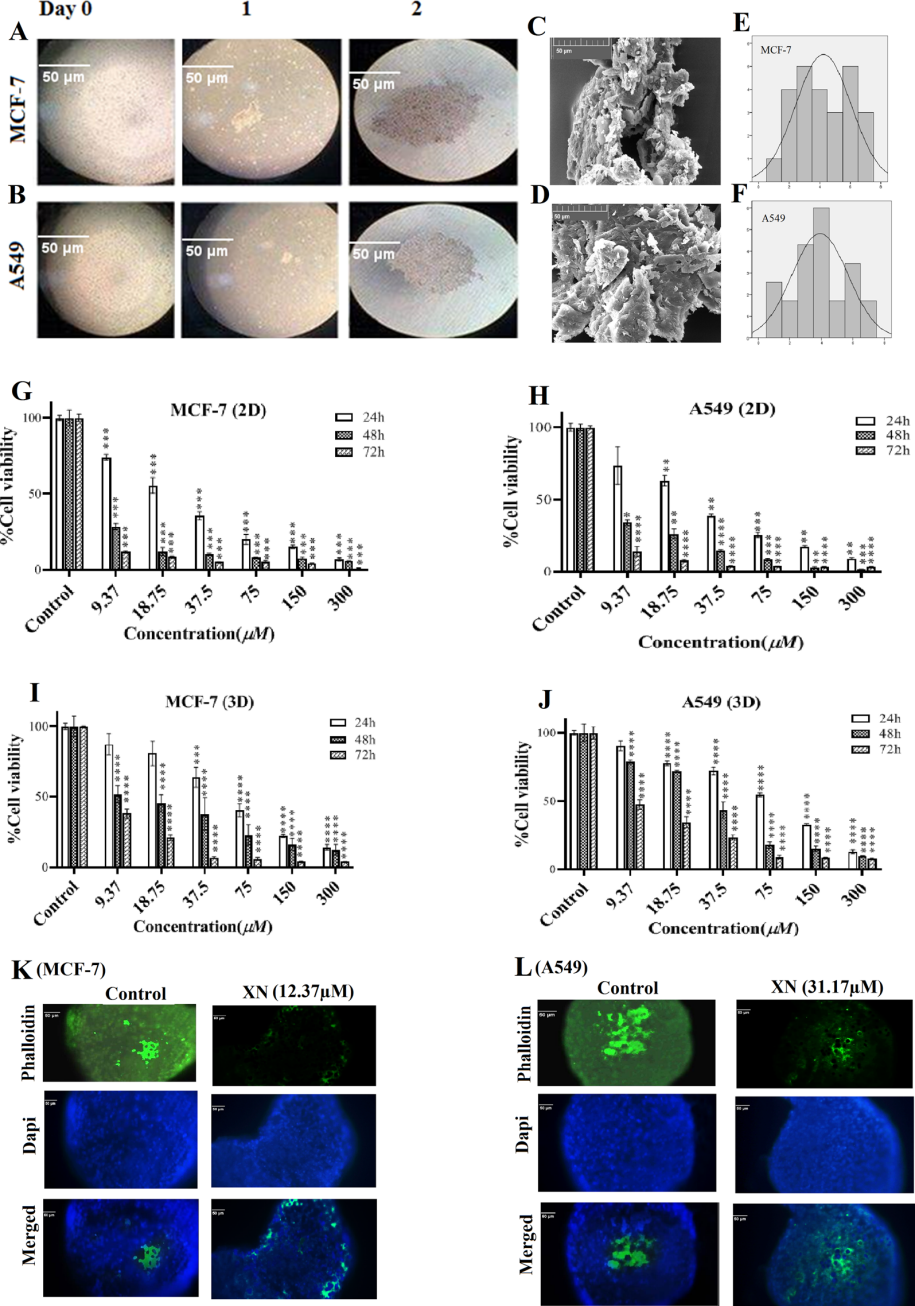
Spheroid formation, inhibitory effects of XN on viability and invasion assay on MCF-7 and A549.** A, B** Shows the images of MCF-7 and A549 spheroids produced using the HD method. Images are taken by inverted microscope at 0, 1, and 2 days (Original magnification 20×) and the scale bar is 50 μm. **C** Shows the SEM photographs of produced spheroids (Original magnification 1000×) and the scale bar is 50 μm. **D** Graphs show the normal size distribution for the produced spheroids in both cell lines. Spheroids were in the range of 98.60 ± 4.25 and 94.17 ± 3.89, for the MCF-7 and A549 cells, respectively. **E**, **F**, **G**, **H** There is a significant difference in the cell viability under 2D and 3D conditions at various times (24, 48, and 72 h) and concentrations (from 9.37 µM to 300 µM).The significant differences comparing the control (i.e., untreated) cells are represented by star(s) as follows: *p < .05, **p < .01, ***p < .001, ****p < .0001). **K**, **L** Microfilaments are decreased in XN-treated MCF-7 **K**, and **L** A549 spheroid

Growth of spheroids was observed (Fig. 2A, B) and verified by an inverted microscope and SEM (Fig. 2C, D). The normality test (mean pixel measure ± SEM) indicated that the population of spheroids had normal distribution for both of the tested cell lines showing 98.60 ± 4.25 and 94.17 ± 3.89 in MCF-7 (Fig. 2E) and A549 (Fig. 2F) cell lines, respectively.

### MTT test for acquiring the XN treatment-IC_50_

The XN treatment-IC_50_ for the MCF-7 cells grown in 2D condition at 24, 48, and 72 h time points were 23.07 µM, 1.9 µM, and 0.18 µM, respectively. In parallel, IC_50_ of the XN treatment of A549 cells were 27.41 µM, 4.74 µM, and 0.48 µM, for the same time points, respectively. However, IC_50_ values for XN were higher when the cells were cultured at 3D conditions. Accordingly, IC_50_ values for the MCF-7 cells were 56.72 µM, 12.37 µM, and 6.20 µM, and for the A549 cells were 77.92 µM, 31.17 µM, and 8.49 µM, at the mentioned three-time points, respectively. Therefore, the IC_50_s at 48 h time point were selected during the cellular and molecular experiments. There were significant differences in the cell viability in different concentrations versus control (Fig. 2G, J).

### XN decreased invasion

Cytoplasmic-filamentous-actin (F-actin) and nuclear-DAPI staining are common methods to test the XN-treatment effect at the cellular level. Microscopic images indicated that XN-treatment caused attenuated actin-microfilaments network intensity in both A549 and MCF-7 spheroids (Fig. 2K, L).

### Flow Cytometry

#### XN arrested cell cycle progression

**Fig. 3:**
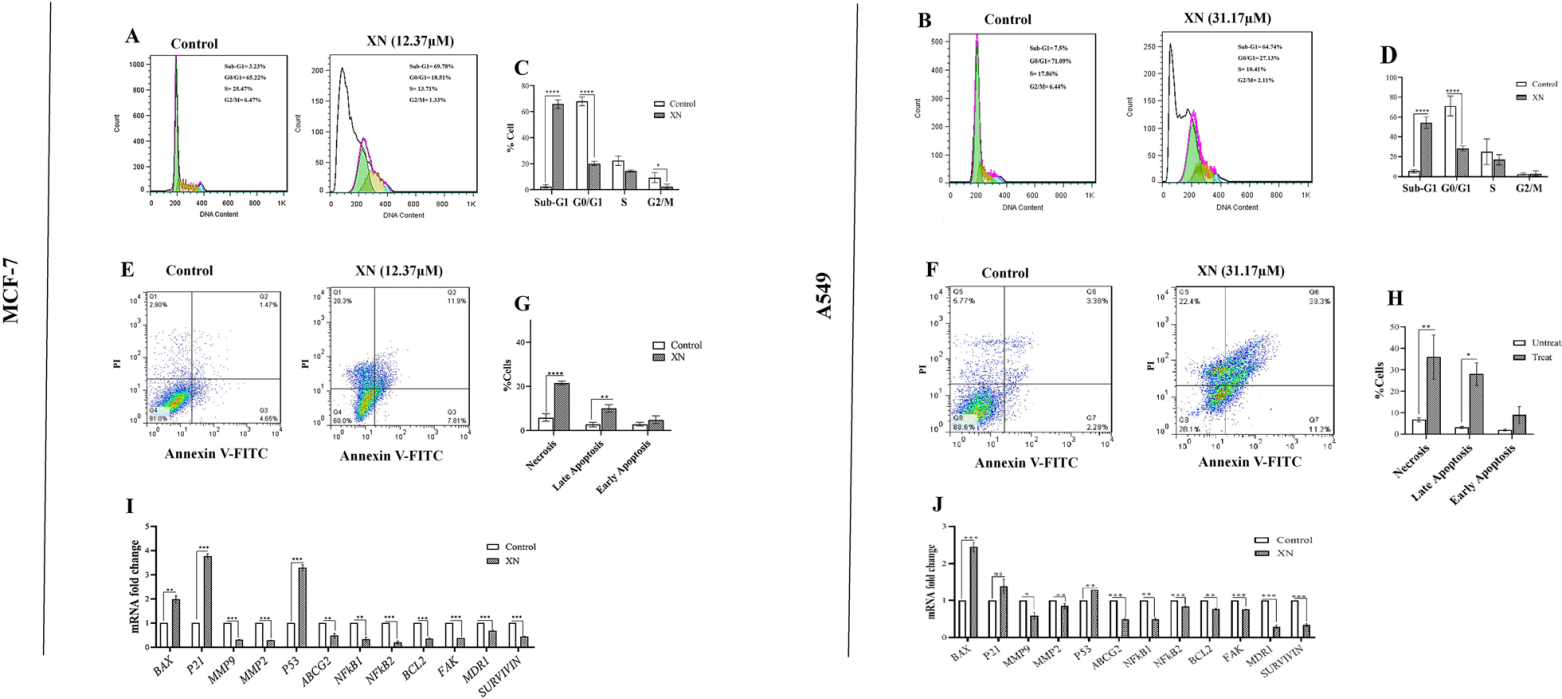
XN-treatment effect on the cell cycle progression, death cell and cancer-related gene expression of MCF-7 and A549 cells. **A**,** B** Flow cytometry results show that the Sub-G1 has been increased, while the G1 cell population has been reduced in the MCF-7 and A549 cells following the treatment. **C**, **D** Representative histogram of cell cycle analysis based on flow cytometry. **E** , **F** Shows Propidium iodide (PI)-Annexin V-FITC binding assay using MCF-7 and A549 cells. Results indicated a significant elevation of necrosis and Late-apoptosis versus **G** XN-untreated-MCF-7 cells and **H** XN-untreated-A549 cells. **I** RT-qPCR results showed that *BAX, P21*, and *P53* genes have been significantly upregulated while the *BCL2* gene has been downregulated following the XN treatment in MCF-7 cells. Also, *MMP9, MMP2, NFKB2, FAK, MDR1*, and *SURVIVIN* genes were significantly downregulated at this condition and *P21*, *P53* genes increased versus control. **J** RT-qPCR results showed that *BAX*, and *P53* genes have been upregulated while other genes *BCL2* gene have been downregulated following the XN-treatment in A549 cells. *P53* genes increased versus control. [All the tests were compared to the untreated control 48 h after treatment with 12.37 µM of XN for MCF-7 and 31.17 µM of XN for A549 and statistical significances are expressed as *p* < .05 (*); *p* < .01 (**); *p* < .001 (***); *p* < .0001 (****)]

XN-treatment of MCF-7 and A549 cells at the chosen IC_50_ resulted in elevated Sub-G1 cell cycle arrest in both cell lines (*p* < 0 001) (Fig. 3C, D). It also brought about the reduced number of the cells in G1 and S phases, detected by flow cytometry (Fig. 3A, B).

#### XN induced apoptosis and necrosis

XN-treatment of MCF-7 and A549 cells also resulted in elevated necrosis, as well as late apoptosis rates in both treated cell lines (Fig. 3E, F). As shown in Fig. 3G, XN treatment resulted in significant induction of late apoptosis (*p* < .001) and necrosis (*p* < .01) in MCF-7. It also promoted late apoptosis (*p* < .01) and necrosis (*p* < .05) in A549 (Fig. 3G, H).

### RT-qPCR

#### Effect of XN treatment on the expression of cancer-related genes

Following the XN treatment of the MCF-7 cells, the expression level of some well-known cancer-related genes was measured through RT-qPCR analysis. Accordingly, *MMP9, MMP2, NFKB2, BCL2, FAK, MDR1*, and *SURVIVIN* genes expressions were significantly reduced (p < .001) while *P21*, and *P53* genes expressions level were increased (p < .001) versus the control. Also, *ABCG2*, and *NFkB1* genes were downregulated (p < .05) while *BAX* gene was upregulated (p < .05) at this condition (Fig. 3I).

In parallel, following the XN treatment of the A549 cells, *NFkB2, FAK, MDR1, SURVIVIN*, and *ABCG2* genes expression levels were reduced (p < .01) while, *BAX* gene expression levels were increased (p < .01). Also, *MMP2, NFkB1*, and *BCL2* gene expressions were decreased p < .05, and *P53* expression level was increased (p < .05) versus control (Fig. 3J).

## Discussion

In this study, the anti-tumor effects of XN on the cell cycle, apoptosis, drug resistance, and invasion in MCF-7 and A549 cancer cell lines are investigated for the first time in a homospheroid culture. XN, a prenylated flavonoid, was extracted from *Humulus lupulus* using maceration method [10] at relatively low temperatures due to the sensitivity of XN to high temperatures. Isolation of XN by P-TLC method showed that 10 mg of XN can be obtained per 10 g of *H. Lupulus* crude extract. Previous studies confirm this result [16], indicating that the P-TLC method is a suitable and high-performance method for the isolation of XN from *H. Lupulus* extract. The structure of XN was confirmed by FT-IR method. Our results were in agreement with the results of the study conducted by Arczewska et al. [17]. Furthermore, the results obtained by FT-IR were confirmed by H-NMR method, consistent with those of Chen et al. [15].

Several studies have confirmed the contribution of XN in apoptosis induction and cell cycle inhibition in cervical cancer, and colon cancer in the monolayer cell culture [3]. These observations can make XN a suitable candidate as an anti-cancer agent in 3D cell cultures.

Spheroids are known as a 3D model for the prediction of drug response in the tumor avascular microenvironment [7]. Studies have shown that compared to 2D cultures, cancer cell lines in 3D cell cultures are less sensitive to drugs, due to the increased cell-cell and cell-ECM interaction [18]. For example, Samimi et al. showed that the IC_50_ for BI-847,325 in the 2D condition is less than the 3D culture of C643 and SW1736 cell lines (Anaplastic thyroid carcinoma) [19]. In another study, it was shown that HTC116 (Human colorectal carcinoma) and H460 (Non-small-cell lung carcinoma) cell lines in 3D conditions are less sensitive to unsymmetrical bisacridines compounds, compared to 2D conditions [20]. Consistent with these reports, the IC_50_ values in our study indicated that the spheroids in 3D conditions are more resistant to XN compounds, compared with the 2D cell cultures. Furthermore, the researchers have shown that drug resistance in 2D conditions is only due to cell-specific mechanisms, while both cell and tissue-specific mechanisms cause this resistance in 3D culture [21]. Various factors play a role in developing resistance in cancer cells [22].

Overexpression of drug efflux pumps is one of the mechanisms that have received more attention. Drug efflux pumps reduce the intracellular concentration of chemotherapy agents in cancer cells [23].

*MDR1* and *ABCG2* genes are two important and key genes in developing resistance in cancer cells. In this study, the effect of XN on these genes was evaluated. Currently, one of the most important reasons for the failure of chemotherapy at invasion and metastasis stage is due to the drug resistance of cancer cells [22]. The discovery of P-glycoproteins has generally been considered as a significant contribution to the ongoing effort to end death and suffering of cancer patients [24]. Although multidrug resistance in cancer cells has several causes, the most important cause is the high expression of P-glycoproteins [25]. P-glycoprotein is an ATP-dependent transporter responsible for drug delivery from the cells, encoded by the *MDR1* gene [26]. The *ABCG2* gene is another cellular pump that we have focused on in this study. The results showed that XN reduces the expression of both *MDR1* and *ABCG2* genes.

In the continuation of this study, the effect of XN treatment on microfilaments’ formation and the expression of important genes involved in the invasion were measured. There is ample evidence that changes in actin polymerization play an essential role in regulating the morphological and phenotypic events of malignant cells. Cancer malignant cells use their innate migration abilities to invade adjacent tissues and arteries and eventually metastasize. Currently, a large number of studies have shown that the molecules that bind migratory signals to the actin cytoskeleton are regulated in invasive and metastatic cancer cells [27]. Our results indicated that treatment of spheroids with XN at the IC_50_ concentration was followed by a reduced level of actin microfilament network, compared to spheroids that were not treated with XN (Fig. 2K L). Matrix metalloproteinases (MMPs) play a pivotal role in cancer progression, especially in the invasion of cancer cells [28]. Focal adhesion kinase (FAK) has been involved in many metastatic processes including adhesion, migration, secretion of MMPs, spindle orientation, and invasion. FAK protein as a tyrosine kinase is overexpressed in cancer cells and has a key role in the development of tumors to a malignant phenotype [29]. Researchers showed that increased mRNA, protein, and FAK activation were directly related to cancer metastasis and invasion [29]. Interestingly, the *MMP2*, *MMP9*, and *FAK* gene expression levels were reduced following the XN treatment (Fig. 3I, J). In order to determine that XN can be a multi-target compound in the treatment of breast and lung cancer cells, in the continuation of the study, we examined the effect of XN on the induction of apoptosis, necrosis and inhibiting the cancer cell cycle both at the cellular and molecular levels.

**Fig. 4:**
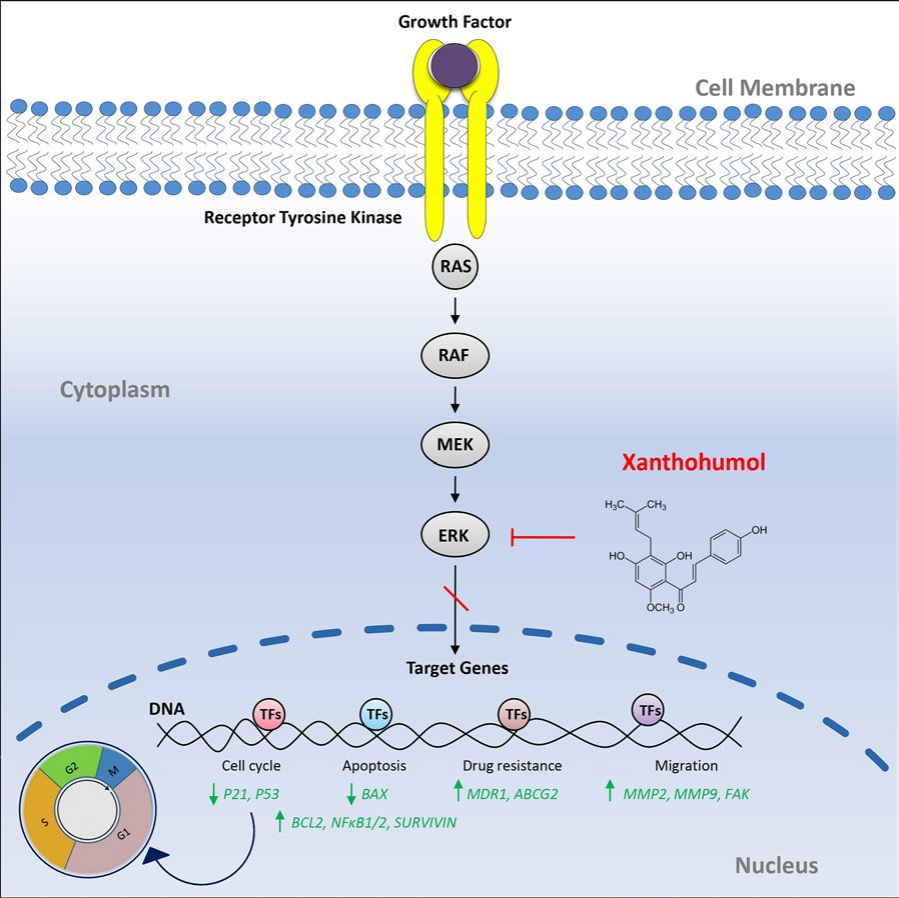
The effect of Xanthohumol on key biological processes associated with breast and lung cancer progression including cell cycle, apoptosis, migration, and drug resistance

Previous studies have shown that XN can inhibit MAPK/ERK pathway [4]. Disruption of the MAPK/ERK pathway is present in many cancers, including breast and lung cancer. The MAPK/ERK cascade through cell surface receptors transmits signals to transcription factors that regulate gene expression and also regulate proteins involved in apoptosis and cell cycle [30] such as *BCL2* [31], *BAX*, *NFk1/2* [32], *P53* [33], *SURVIVIN* [34] (Fig. 4).

One of the apoptotic pathways that MAPK/ERK regulates is the Bcl-2/Bcl-xL/Bax signaling pathway. Bcl-2/Bcl-xL/Bax and MAPK/ERK pathways are frequently dysregulated in many human cancers. Observations suggest that both MAPK and BCL-2/BAX signaling play a central role in human cancer pathogenesis [31].

Flow cytometry results indicated that cell cycle has been arrested at the Sub-G1 phase following the XN treatment of the MCF-7 and A549 cell lines. Also, these treatments induced necrosis and apoptosis in these cells. It has been reported that cancer cells may escape from drugs through the cell cycle phase-specific drug resistance. The induction of apoptosis is one of cancer treatment strategies in various cancer cells. However, this attitude does not evoke a therapeutic consequence in apoptosis-resistant tumors. To solve this problem, the induction of necrosis has been suggested as a new strategy against apoptosis-resistant tumor cells [35]. Yin et al. showed that XN stopped cell cycle in esophageal cancer cells. They also showed that it reduces *BCL2* expression and induces *BAX* gene expression. The arrest of the treated MCF-7 and A549 cells with XN seems consistent with these reports.

At the molecular level, XN treatment of the cells was followed by reduced *NFĸB1/2* expression level, which is consistent with the report by Hinz et al. that provided evidence for a direct link between NFĸB and cell cycle regulation [36]. Also, reports have emphasized that each BCL-2/BAX signaling pathway plays a major role in the pathogenesis of human cancer [31]. Our results indicated that apoptosis, as well as necrosis, have been induced through the reduced expression level of *NFĸB1, NFĸB2, SURVIVIN*, and *BCL 2* genes following the treatment with XN. Liu et al. have shown that XN treatment of the HT-29 cells (Intestinal cancer cell rejection) resulted in decreased *BCL2* expression and increased *BAX* expression under 2D culture [37]. *SURVIVIN* as a member of the apoptosis inhibitory protein family, which might hinder caspases and inhibit cell death, is highly expressed in most cancers and has poor clinical outcomes [38].

The P53 is a superstar in suppressing tumorigenesis and the high incidence of mutations in this gene in tumor cells suggests the P53 pathway is a promising treatment target for cancer. On the other hand, a large number of studies have shown that activated MAP kinase signaling pathway increases the level of *P53* mRNA [33]. As a result, there are various treatment strategies that target the *P53* pathway [39]. Yong et al. showed that XN can induce *P53* expression in CaSk cell line (cervical cancer cell line) under 2D cell culture conditions [40]. The present study also showed that XN can induce the *P53* gene expression in A549 and MCF-7.

Contrary to the previous studies which have highlighted the anti-cancer effects of xanthohumol in 2D cell culture, this research is focused on investigating the effect of xanthohumol on 3D cell culture. The results of 3D culture experiments have higher comparability between in vitro and human trials. On the other hand, it is possible to decrease the use of in vivo tests by minimizing the need to test directly on animals. However, 3D cell culture cannot be completely replaced with animal use [41].

## Conclusion

This study highlights the anti-cancer activities of XN, a natural bioactive compound derived from *Humulus lupulus*, against breast and lung cancer cells in 3D cell culture. The IC_50_s of the hemispheroid model showed that this model is more resistant to XN than the 2D model. Particularly, XN is shown to induce apoptosis and cell cycle arrest, inhibition of invasion, and reduction of resistance of lung and breast cancer cells.

According to the results obtained in this study, the hemospheroid model designed in this study has provided an opportunity for future studies to investigate the effect of XN and other bioactive compounds on various signaling pathways in breast and lung cancer. By performing additional tests including laboratory animal study and in breast and lung cancer, XN can be used as a multi-target adjuvant natural compound in the treatment of cancer cells.

## Data Availability

The datasets generated and/or analyzed during the current study are available from the corresponding author on reasonable request.

## Abbreviations

2D: Two-dimensional
3D: Three-dimensional
ABCG2: ATP-binding cassette subfamily G member 2
BAX: Bcl-2-associated X protein
BCL2: B-cell leukemia/lymphoma 2 protein
DMEM: Dulbecco’s modified eagle’s medium
F-actin: Cytoplasmic-filamentous-actin
FAK: Focal adhesion kinase
FBS: Fetal bovine serum
FTIR: Fourier transform infrared spectroscopy
H-NMR: Hydrogen nuclear magnetic resonance
HD: Hanging drop
IC_50_: Half-maximal inhibitory concentration
MAPK/ERK: Kinase/ extracellular signal-regulated Kinase
MP2: Matrix metalloproteinase 2
MMP9: Matrix metalloproteinase 9
MDR1: ATP binding cassette Subfamily B Member 1
NF*κ*B1: Nuclear factor kappa B subunit 1
NF*κ*B2: Nuclear factor kappa B subunit 2
P21: Cyclin dependent kinase inhibitor 1 A
P53: Tumor protein P53
P-TLC: Preparative-thin layer chromatography
PBS: Phosphate-buffered saline
qRT-PCR: Quantitative real-time PCR
SEM: Scanning electron microscope
TFs: Transcription factors
XN: Xanthohumol
